# The Assembly of the Plasmodial PLP Synthase Complex Follows a Defined Course

**DOI:** 10.1371/journal.pone.0001815

**Published:** 2008-03-19

**Authors:** Ingrid B. Müller, Julia Knöckel, Matthew R. Groves, Rositsa Jordanova, Steven E. Ealick, Rolf D. Walter, Carsten Wrenger

**Affiliations:** 1 Department of Biochemistry, Bernhard Nocht Institute for Tropical Medicine, Hamburg, Germany; 2 European Molecular Biology Laboratory-Hamburg Outstation, Hamburg, Germany; 3 Department of Chemistry and Chemical Biology, Cornell University, Ithaca, New York, United States of America; Institute of Molecular and Cell Biology, Singapore

## Abstract

**Background:**

Plants, fungi, bacteria and the apicomplexan parasite *Plasmodium falciparum* are able to synthesize vitamin B6 *de novo*, whereas mammals depend upon the uptake of this essential nutrient from their diet. The active form of vitamin B6 is pyridoxal 5-phosphate (PLP). For its synthesis two enzymes, Pdx1 and Pdx2, act together, forming a multimeric complex consisting of 12 Pdx1 and 12 Pdx2 protomers.

**Methodology/Principal Findings:**

Here we report amino acid residues responsible for stabilization of the structural and enzymatic integrity of the plasmodial PLP synthase, identified by using distinct mutational analysis and biochemical approaches. Residues R85, H88 and E91 (RHE) are located at the Pdx1:Pdx1 interface and play an important role in Pdx1 complex assembly. Mutation of these residues to alanine impedes both Pdx1 activity and Pdx2 binding. Furthermore, changing D26, K83 and K151 (DKK), amino acids from the active site of Pdx1, to alanine obstructs not only enzyme activity but also formation of the complex. In contrast to the monomeric appearance of the RHE mutant, alteration of the DKK residues results in a hexameric assembly, and does not affect Pdx2 binding or its activity. While the modelled position of K151 is distal to the Pdx1:Pdx1 interface, it affects the assembly of hexameric Pdx1 into a functional dodecamer, which is crucial for PLP synthesis.

**Conclusions/Significance:**

Taken together, our data suggest that the assembly of a functional Pdx1:Pdx2 complex follows a defined pathway and that inhibition of this assembly results in an inactive holoenzyme.

## Introduction

The active form of vitamin B6 is pyridoxal 5-phosphate (PLP), which is an essential cofactor for more than 100 enzymes and thereby involved in catalytic reactions such as amino acid decarboxylation, elimination and amino-transfer [1]. PLP is synthesized *de novo* by plants, almost all bacteria and fungi; however, mammals depend entirely on the uptake of this indispensable nutrient from their diet. As shown for yeast, the non-phosphorylated inactive cofactor is imported via specific transporters and finally trapped within the cell by phosphorylation catalyzed by pyridoxal kinase (PdxK) [2], [3]. Thus, the dual provision of PLP by *de novo* synthesis and salvage indicates necessity and importance of this cofactor for the survival of yeast and other organisms.

**Figure 1:**
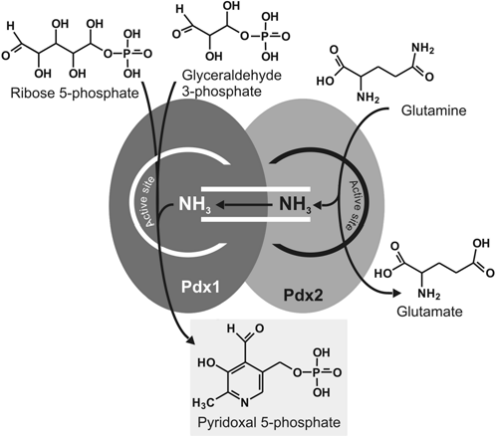
Reaction scheme of the Pdx1 and Pdx2 proteins. The *Pf*Pdx2 enzyme depends on its interaction with *Pf*Pdx1 for glutaminase activity. The Pdx1 enzyme is responsible for the *de novo* synthesis of PLP by utilizing the substrates ribose 5-phosphate, glyceraldehyde 3-phosphate and ammonia, whereas the latter is provided by deaminating of glutamine by Pdx2 via substrate channelling to the attached *Pf*Pdx1 protein. The reactions of each enzyme are indicated.

Currently two different pathways for the biosynthesis of PLP are known. The *E. coli*- (DOXP-dependent) creates from the substrates 4-phosphohydroxy-L-threonine, 1-deoxyxylulose 5-phosphate (DOXP) and glutamate pyridoxine. In contrast the fungi-like- (DOXP-independent) pathway, which has been firstly described in the fungus *Cercospora nicotianae*, synthesises the active cofactor PLP from ribose 5-phosphate, glyceraldehyde 3-phosphate and glutamine by an enzyme complex consisting of two proteins - Pdx1 and Pdx2 [4]–[11]. The Pdx2 protein exhibits glutaminase activity and delivers ammonia to Pdx1 [5], [6] (Fig. 1). The crystal structures of Pdx1 (YaaD or PdxS), and Pdx2 (YaaE or PdxT) from various organisms have been analyzed [12]–[14]. Only recently the structures of the B6 biosynthesis complex (PLP synthase) from *T. maritima* and *B. subtilis* have been solved. Pdx1 assembles into a dodecamer, consisting of two hexameric crowns, each decorated by six Pdx2 molecules [15], [16].

Here we report on biochemical analyses of the interaction of the plasmodial Pdx1:Pdx1 interfaces as well as about effects on Pdx2 binding and catalysis. In contrast to the static nature of the Pdx1 crystal structures, this study allows functional insights into the behaviour of the highly dynamic plasmodial complex and suggests a possible path for its assembly. The amino acids that are crucial for the plasmodial PLP complex should be further exploited for the design of specific drugs which will be restricted to the malaria parasite and not harming the human host.

Malaria is one of the most serious infectious diseases in the world (WHO, Communicable Disease Report). Antimalarial drugs are losing more and more efficacy against the deadliest agent, *Plasmodium falciparum*. Since a vaccine is not available, an urgent need exists to identify novel targets for the development of new chemotherapeutics [17].

## Results

### Specific activity and mutagenic analyses of the active sites of Pdx1 and Pdx2

The specific activity of the plasmodial vitamin B6 biosynthesis complex consisting of *Pf*Pdx1 and *Pf*Pdx2 (1∶1 ratio) was determined to be 662±54 pmol min^−1^ mg^−1^ protein if ribose 5-phosphate, glyceraldehyde 3-phosphate and glutamine were used as substrates. This is about seven-fold higher than the specific activity previously observed for the plasmodial enzymes by Gengenbacher *et al*. [14] and might result from a different expression system and purification methods. In the presence of ammonium chloride instead of glutamine, the plasmodial Pdx1 protein alone revealed a specific activity of 746±76 pmol min^−1^ mg^−1^ protein. Because of the observed similar activity, analyses on the amino acid residues from the *Pf*Pdx1 active site as well as for the Pdx1:Pdx1 interface were carried out by the latter enzyme assay without *Pf*Pdx2.

**Figure 2:**
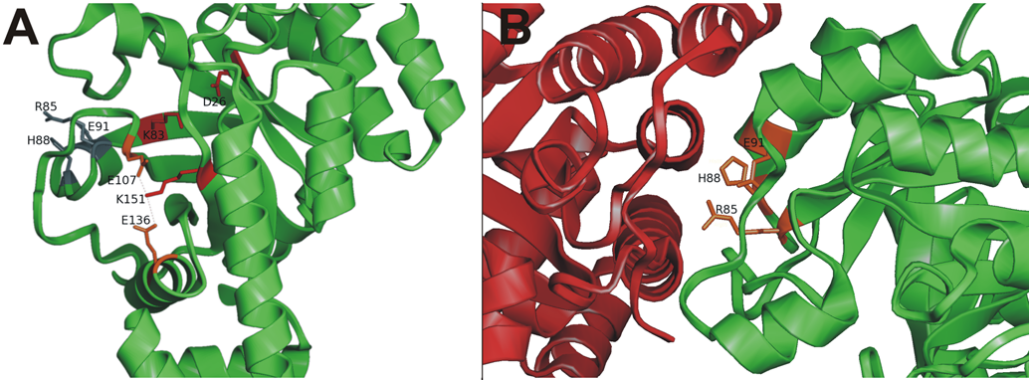
(A) Homology model of one plasmodial Pdx1 monomer showing the analyzed amino acid residues as indicated. (B) The interface region between two *Pf*Pdx1 proteins within the same hexameric ring illustrating the amino acid residues R85, H88 and E91, which are involved in Pdx1:Pdx1 binding. The model was generated by Swiss-Model [30] and visualised by PyMOL (www.pymol.org).

**Figure 3:**
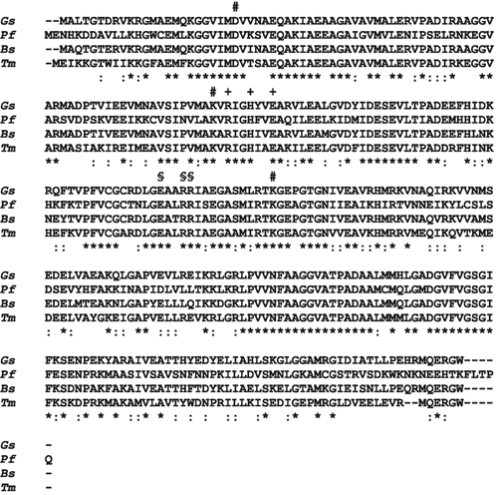
Alignment of the deduced amino acid sequences of the YaaD protein from *Thermotoga maritima* (*Tm,* AC: Q9WYU4), the Pdx1 protein from *Bacillus subtilis* (*Bs,* AC: NP_387892), the PdxS protein from *Geobacillus stearothermophilu*s (*Gs,* AC: Q5L3Y2) with the Pdx1 protein from *P. falciparum* (*Pf,* AC: XP_966196). The alignment was performed by using CLUSTALW [31]. Identical (*) and similar (:) amino acid residues are indicated below the protein sequence. Gaps (-) were introduced into the sequence to maximize homology and to compensate for the different chain lengths. Mutated amino acid residues, which are predicted to be participating within the active site and the additional phosphate binding site, are labelled above by (+) and (§), respectively. The exchanged amino acid residues, proposed to be involved in the Pdx1:Pdx1 interaction, are labelled above the sequence by (#).

**Figure 4:**
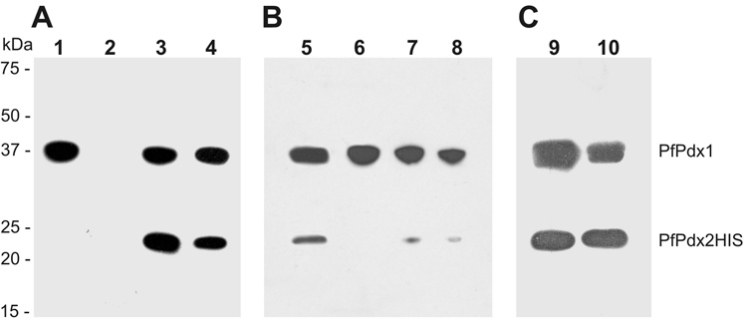
Western blot analysis of the co-purified *Pf*Pdx1 and *Pf*Pdx2HIS proteins. Cell homogenates of the recombinant expression of the plasmodial Pdx1 protein and Pdx2HIS protein (6× His-tag instead of a Strep-tag) were mixed and subsequently purified via Strep-tag affinity chromatography of the *Pf*Pdx1 protein. The co-purification was visualized by Western blot analysis using a monoclonal anti-Strep antibody (IBA) and the HIS-probe-HRP (Pierce) as described in Material and Methods. (A) Co-purification of the *Pf*Pdx1 wild-type (3) and the *Pf*Pdx1 DKK (D26A, K83A and K151A) triple mutant protein (4) with the *Pf*Pdx2HIS wild-type. As a control the *Pf*Pdx1 wild-type (WT) (1) and the *Pf*Pdx2HIS (2) single proteins were purified via Strep-tag affinity chromatography and applied in Western blot analysis. (B) Co-purification of the *Pf*Pdx1 ERR (5), *Pf*Pdx1 RHE (6) as well as the *Pf*Pdx1 H88A (7) and *Pf*Pdx1 E91A (8) mutant proteins with the plasmodial Pdx2HIS wild-type. (Note: The *Pf*Pdx1 R85A mutant was not recombinantly expressible.) (C) The gate-keeper *Pf*Pdx2HIS E53Y (9) and *Pf*Pdx2HIS R154W (10) mutants were co-purified with the *Pf*Pdx1 wild-type protein via its Strep-tag.

Structural analysis of Pdx1 (PdxS) from *Geobacillus stearothermophilu*s suggested a participation of residues D24, K81 and K149 in the binding of ribulose 5-phosphate or catalysis [13]. Subsequently the amino acids K149 from *B. subtilis* and K82 in the PLP synthase subunit (YaaD) from *Thermatoga maritima*, respectively, were observed to be covalently attached to ribulose 5-phosphate [7], [15]. The homologues of these residues in Pdx1 of the plasmodial enzyme are D26, K83 and K151, respectively (Fig. 2A). These amino acid residues were substituted by alanine using site directed mutagenesis and the mutant proteins were analysed for enzymatic activity (Fig. 3). The *Pf*Pdx1 DKK (D26A/K83A/K151A) triple mutant enzyme as well as each individual mutant were inactive. The results confirmed that residues D26, K83 and K151 are important for *Pf*Pdx1 activity; however, a glutaminase assay with the *Pf*Pdx2 wild-type enzyme and the respective *Pf*Pdx1 showed that the ability of Pdx2 to hydrolyze glutamine was not affected with activities in the same range as previously reported for the wild-type enzymes [6] (Table 1). This indicates that the *Pf*Pdx1 DKK mutant still binds to *Pf*Pdx2. To verify the ability of the *Pf*Pdx1 DKK mutant to bind to the C-terminal 6× HIS-Tag harbouring *Pf*Pdx2HIS wild-type protein, co-purification analyses were performed (Fig. 4A). The results clearly demonstrate that modification of these active site residues of *Pf*Pdx1 does not affect its ability to interact with *Pf*Pdx2.

**Table 1:** Properties of *P. falciparum* mutated Pdx1 and Pdx2 proteins in comparison to the wild type.

Mutations in *Pf*Pdx1	*Tm*YaaD	*Pf*Pdx1	*Pf*Pdx2	Oligomerization state of *Pf*Pdx1	Pdx1:Pdx2 interaction
		[µU mg^−1^]	[mU mg^−1^]		
Wild-type (WT)		746±76	266±19	dodecamer	+
D26A	25	n. d.	285±30	dodecamer	+
K83A	82	n. d.	291±22	dodecamer	+
K151A	150	n. d.	251±16	hexamer	+
DKK to AAA (D26/K83/K151)	25, 82, 150	n. d.	274±44	hexamer^a^	+
H88A	87	n. d.	172±13	monomer	(+)
E91A	90	n. d.	95±14	monomer	(+)
RHE to AAA (R85/H88/E91)	84, 87, 90	n. d.	n. d.	monomer	-
ERR to AAA (E136/R139/R140)	135, 138, 139	n. d.	286±8	dodecamer	+

The properties of the recombinant *P. falciparum* vitamin B6 biosynthesis enzymes Pdx1 and Pdx2 were determined as described in the Material and Methods section. The results represent the mean values of at least three independent experiments; (*Tm*YaaD) corresponding amino acid residues of YaaD (Pdx1) in *T. maritima*; (*Pf*Pdx1) PLP formation by *Pf*Pdx1 (pmol min^−1^ mg^−1^); (*Pf*Pdx2) glutaminase activity of *Pf*Pdx2 (nmol min^−1^ mg^−1^); (+) co-purification of *Pf*Pdx1 and *Pf*Pdx2 to a lesser extent; n. d. = not detectable for PLP formation by *Pf*Pdx1 (<10 pmol min^−1^ mg^−1^).

a approx. 50% dodecamer in the presence of 10 mM glutamine

### K151 is crucial for the dodecameric conformation of the plasmodial Pdx1

**Figure 5:**
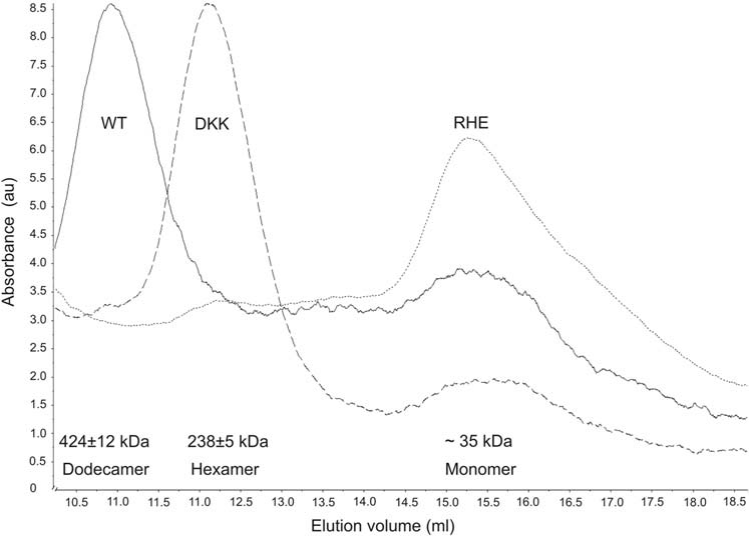
Static light scattering of the plasmodial Pdx1 DKK and RHE mutant proteins in comparison to the wild-type (WT). In order to analyze the structural conformation of the *Pf*Pdx1 proteins, size exclusion chromatography was performed with the *Pf*Pdx1 WT (—), *Pf*Pdx1 DKK (----) and the *Pf*Pdx1 RHE (……) mutant proteins and the masses measured by static light scattering. The corresponding molecular masses and the proposed structural assemblies are given below. Aliquots of the three peak fractions were subsequently analysed by SDS-PAGE, which confirmed the presence of *Pf*Pdx1 (data not shown).

The molecular mass of the wild-type *Pf*Pdx1 dodecamer is 424±12 kDa, as determined by static light scattering (SLS) (Fig. 5). These data indicate that the wild-type *Pf*Pdx1 assembles into a dodecameric structure (double crown formation), as has been shown for the counterparts in *T. maritima* and *B. subtilis* 15, 16. Surprisingly, the double crown formation is inhibited in the *Pf*Pdx1 DKK mutant, which falls apart into two separate crowns, thereby revealing a hexameric structure with molecular mass of 238±5 kDa as determined by SLS (Fig. 5). In order to narrow down which amino acid residues of the *Pf*Pdx1 DKK triple mutant are involved in *Pf*Pdx1:*Pf*Pdx1 interactions, the three amino acids were mutated individually and the derived proteins were analysed by SLS. Mutation of the *Pf*Pdx1 D26 and K83, which are expected to lie within the active site, does not alter the assembly state of *Pf*Pdx1 wild-type protein; however the mutation of *Pf*Pdx1 K151 to alanine - as shown for the DKK triple mutant in Figure 5 - destabilizes the *Pf*Pdx1 assembly (Table 1). The result indicates that this amino acid residue, previously suggested to participate in the formation of the active site of *Pf*Pdx1, is also involved in the formation of the dodecameric structure of Pdx1 in *P. falciparum*.

**Figure 6:**
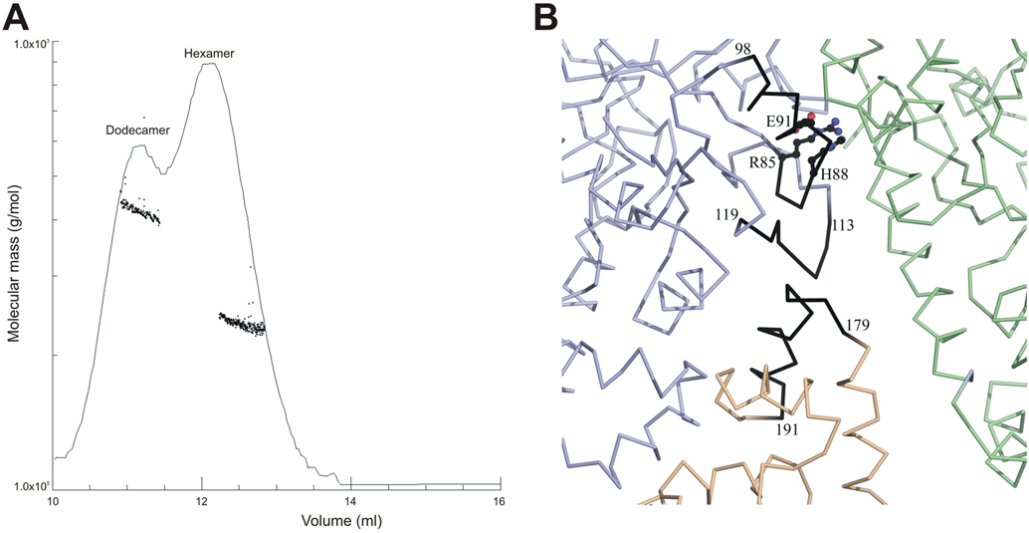
(A) Influence of glutamine on the conformation of the *Pf*Pdx1 DKK mutant. Static light scattering analysis of the plasmodial Pdx1 DKK protein was performed in the presence of 10 mM glutamine. The proposed structural conformations are given above the peak fractions. (B) Affect of mutations on Pdx1 interactions between hexamers. Monomers coloured in green and violet belong to the upper hexameric crown and the monomer coloured in salmon belongs to the lower hexameric crown. Mutations in R85, H88 and E91 of the upper crown affect the conformation of loop 113–119, which interacts with loop-helix 179–191 in the lower crown. Helix 88–98 interacts directly with Pdx2 (not shown).

Interestingly, addition of 10 mM glutamine (Q) to the buffer used in size exclusion chromatography, resulted in an approximate 50% shift in the assembly of the hexameric formation of the DKK mutant towards the dodecameric structure as estimated from the molecular mass plot obtained by SLS (Fig. 6A). A similar effect was obtained by the addition of 10 mM glutamic acid (E) as well as asparagine (N), although to a lesser extent. In contrast alanine (A) does not affect dodecamer formation.

The amino acid residues E107, E136 and K189 were identified as being proximal to K151 using a homology model of the plasmodial Pdx1 protein (Fig. 2A). E107 and E136 were mutated to alanine and analyzed for their conformation by size exclusion chromatography and SLS. None of these mutants, including the double mutation of both glutamic acids, E107 and E136, showed an effect on dodecamer assembly (data not shown).

Previous crystal structure analyses showed that H116, R131, E135, R138, R139, K150 and K188 in the *T. maritima* structure are located in a phosphate-binding site [15]. All of these amino acid residues are conserved in the plasmodial Pdx1 protein at the positions H117, R132, E136, R139, R140, K151 and K189 (Fig. 3). Mutating the amino acid residues E136, R139 and R140 to alanine (ERR) abolishes the enzyme activity of *Pf*Pdx1, implying the importance of these residues for PLP synthesis. Nevertheless, the *Pf*Pdx1 ERR mutant reveals a molecular mass of 421±20 kDa as measured by SLS, which correlates well with the dodecameric formation of the wild-type complex. The glutaminase activity of *Pf*Pdx2 is not affected (Table 1), indicating binding of Pdx2 to Pdx1 as confirmed by co-purification experiments (Fig. 4B).

As a result of these experiments, we propose that the role of K151 in the assembly of dodecameric Pdx1 does not depend upon the neighbouring residues in the final assembled structure, rather that its interaction partners during the assembly must be distal from its final position, possibly even on adjacent monomers of the dodecamer.

### Analysis of the interaction of Pdx1 monomers in P. falciparum

As already mentioned, biochemical analyses of the plasmodial PLP synthase revealed a 1∶1 ratio of both Pdx1 and Pdx2 proteins [6], [14]. The crystal structures of the PLP synthase of *T. maritima* and *B. subtilis* demonstrate that the dodecameric complex of Pdx1 is decorated by the identical number of Pdx2 monomers, which in turn have no direct contact to one another and are therefore interacting only with the respective Pdx1 protein [15], [16]. Thus, the double crown complex depends solely on the interaction of Pdx1. Through an analysis of the protein sequence of the plasmodial Pdx1 and the homology model, a number of amino acids within the *Pf*Pdx1:*Pf*Pdx1 interface region were identified (Fig. 2B). These residues include R85, H88 and E91 in *P. falciparum*, corresponding to amino acids conserved in other organisms as indicated in Figures 2A,B and 3. To analyse the impact of these residues on enzyme activity as well as their importance for the assembly of the entire complex, they were mutated to alanine. No PLP synthesis activity of the plasmodial Pdx1 mutants was obtained; however, the glutaminase activity of *Pf*Pdx2 remained, although it was reduced to approx. 66% and 36% in the presence of the *Pf*Pdx1 mutants H88A and E91A, respectively (Table 1). The reaction catalyzed by *Pf*Pdx2 complexed with the *Pf*Pdx1 triple mutant RHE did not reveal any detectable glutaminase activity, indicating that the interaction of the *Pf*Pdx1 monomers might affect *Pf*Pdx2 binding. Furthermore, to verify whether the homomeric relations influence the assembly of *Pf*Pdx1 and *Pf*Pdx2, co-purification experiments were performed. The *Pf*Pdx1 wild-type protein and mutants as well as the *Pf*Pdx2HIS wild-type protein were heterologously expressed and the bacterial homogenates were mixed in an approx. 1∶1 ratio. Subsequently, the proteins were purified via affinity chromatography using the Strep-tag of the respective *Pf*Pdx1 wild-type or mutant proteins. Western blot analysis clearly demonstrated co-purification of the wild-type forms, whereas the *Pf*Pdx1 mutants were found to be incompetent in binding *Pf*Pdx2 (Fig. 4B). The oligomeric states of the *Pf*Pdx1 RHE, H88A and E91A mutant proteins were further analyzed by size exclusion chromatography and SLS. No significant assembly to dodecameric or hexameric formation was detected in either the absence (Fig. 5/6A) or presence of glutamine. Therefore, the *Pf*Pdx1 RHE triple mutant impedes formation of the entire complex and exists in solution only as a monomer. Furthermore, even the single mutants H88A and E91A led to a monomeric state. The R85A mutant did not express and no further analysis of this mutant was performed.

### Ammonia shuttle from PfPdx2 to PfPdx1

PLP synthesis by *Pf*Pdx1 depends on ammonia, which is supplied by the hydrolysis of glutamine to glutamate and ammonia by *Pf*Pdx2, the glutaminase attached to *Pf*Pdx1 [6], [14]. Ammonia is thought to be transferred to the catalytic site of Pdx1 via a protein channel [13], [15], [16]. Analysis of the Pdx2 structure led to the suggestion that the amino acid residues E53 and R154 are the gate-keepers of the ammonia translocation to Pdx1 [15]. Exchanging these amino acids to tyrosine (E53Y) and tryptophan (R154W), respectively, resulted in the total loss of the *Pf*Pdx2 glutaminase activity, while the ability of *Pf*Pdx1 to generate PLP is not affected if ammonium is added as an ammonia source. These mutations did not influence the ability of *Pf*Pdx2 to form a complex with *Pf*Pdx1 as clearly demonstrated by co-purification experiments (Fig. 4C).

## Discussion

Two different vitamin B6 biosynthetic pathways have been previously described [18], [19]. While the DOXP-dependent pathway, consisting of the Pdx protein family, has been well characterised [10], [11], [20], [21], the DOXP-independent pathway, first found in fungi, has only recently been identified [18], [19]. The enzymes involved in the DOXP-independent pathway share neither sequence similarities nor substrate acceptance with *E. coli* Pdx proteins. Furthermore, vitamin B6 biosynthesis in *E. coli* leads to pyridoxine phosphate, which is subsequently oxidized to PLP by pyridoxine oxidase. The synthesis of the DOXP-independent pathway seems to be more efficient since it results directly in the formation of pyridoxal phosphate, the active form of vitamin B6. This pathway, originally identified by Ehrenshaft *et al.* [22] in *Cercospora nicotianae*, a plant pathogen, is also found in *S. cerevisiae* as well as some archaebacteria, eubacteria, the plant *A. thaliana*, and recently in the protozoan parasites *P. falciparum* and *T. gondii* [5], [6], [9], [18], [23].

PLP is an essential cofactor for various enzymes many of which are involved in fundamental metabolic reactions [1]. Deletion of vitamin B6 biosynthesis enzymes resulted in auxotrophy for this nutrient in bacterial cells [24] and led to a developmental arrest in plant embryos [9]. Attempts to disrupt the open reading frame of the plasmodial *pdx1* have failed so far, which might indicate an indispensability of the gene for the survival of the parasite.

Analyses of the crystal structures of the entire PLP synthase complex in *T. maritima* and *B. subtilis* revealed a dodecameric Pdx1 conformation, which is decorated by twelve Pdx2 proteins [15], [16]. Very recently, the interface of Pdx1 and Pdx2 has been described [16]; however, amino acid residues involved in the interaction of the Pdx1 subunits to form the double crown remained to be explored. Therefore, site directed mutagenesis was employed to modify highly conserved amino acid residues that were suggested to form a buried charge cluster and might be located in the Pdx1:Pdx1 interface [13], [15]. These highly conserved residues are R85, H88, E91 and D222 in the plasmodial Pdx1 enzyme. Substitution of H88 and E91 by alanine as well as the derived triple mutant RHE (R85A, H88A, E91A) resulted in the loss of *Pf*Pdx1 activity. Interestingly, these mutant proteins were neither able to form a dodecamer nor to associate with *Pf*Pdx2. While these residues primarily interact within the hexameric crown, they interact indirectly with the opposing hexamer through loop 113–119. This loop in turn contacts helix 179–191 of the second hexamer and may mediate dodecameric formation through this mechanism (Fig. 6B). The effect of RHE on Pdx1-Pdx2 interactions is less clear. These residues are positioned at the N-terminus of helix 88–98 and the loop following this helix interacts directly with Pdx2; thus, it is possible that R85, H88 and E91 are required indirectly for Pdx1-Pdx2 interactions as well.

In *T. maritima* the active site of YaaD contains the amino acid residues D25, K82 and K150 [15]. Mutation of these residues in the bacterial Pdx1 enzyme impedes PLP synthesis [15], [25]. This motif is also present in the plasmodial Pdx1 protein and mutagenic analyses of the respective residues (D26A, K83A and K151A) show loss of Pdx1 activity. However, the mutations have no impact on the *Pf*Pdx1:*Pf*Pdx2 binding ability and on the glutaminase activity of *Pf*Pdx2 (Table 1). Interestingly, the *Pf*Pdx1 DKK mutation leads to a split of the dodecamer into two hexamers. In the presence of glutamine, the destabilizing effect of the DKK triple mutation is reduced by about 50% suggesting an involvement of glutamine in the stabilization of the *Pf*Pdx1 assembly.

The dissociation of the dodecamer is demonstrated to be a result of the K151A mutation. Examination of the structural data of the *B. subtilis* Pdx1 complex (2NV2; www.pdb.org) demonstrates that the equivalent residue (K149) orientates such that the terminal amino group points away from the active site and towards the central cavity of the assembled Pdx1 dodecamer. Nevertheless, it has been shown that K149 in the *B. subtilis* enzyme is involved in ribulose 5-phosphate binding [7]. K151 in the homologous *P. falciparum* Pdx1 is located in neither the Pdx1 nor the Pdx2 interface and consequently its role in formation of the Pdx1 dodecamer is intriguing. In the structure of *T. maritima* YaaD, this residue is pointed towards the phosphate binding site containing ERR [15]. It has been proposed that this site marks the glyceraldehyde 3-phosphate binding site or an alternative ribulose 5-phosphate binding site and that K151 participates in this binding [15]. ERR directly interact with helix 183–191 of the opposing hexameric crown forming extensive interactions. Interestingly, addition of exogenous amino acids with similar sizes and/or charge to K151 can mimic the function of this lysine by partially restoring the dodecameric conformation. However, this result was observed *in vitro* and remains for elucidation whether this effect is of physiological relevance.

The observation that Pdx1 exists as monomers, hexamers and dodecamers, and that only the hexamers and dodecamers interact with Pdx2 suggests a possible order of assembly. Hexameric crowns probably form first, likely mediated by residues R85, H88 and E91. Once a crown has formed, it can bind Pdx2, as evidenced by the data from our co-purification assays, followed by the assembly of two crowns to form a dodecameric Pdx1 core. Alternatively, the dodecameric core could form first, followed by addition of 12 Pdx2 monomers. These two possibilities are not easily differentiated and clarification of the exact mechanism will require additional biochemical and structural studies.

It has been reported that the bacterial YaaD and PdxS proteins from *T. maritima* and *G. stearothermophilus*, respectively, possess an additional phosphate binding site, which might interact with the phosphate group of the second substrate, glyceraldehyde 3-phosphate [13], [15]. Modification of the homologous amino acid residues in *P. falciparum*, E136, R139 and R140, abolished *Pf*Pdx1 activity, which emphasizes the important role for catalysis of these amino acid residues.

For the generation of the active cofactor PLP, an ammonia source is required, which is provided by the glutaminase Pdx2 via substrate channelling [6], [14], [23]. Structural analysis of the bacterial YaaD (Pdx1) and YaaE (Pdx2) complexes suggested a putative ammonia channel [15], [16]. The passage of ammonia seems to be modulated by a gate consisting of E47 and R135 in the *T. maritima* enzyme [15]. Mutagenesis of the respective conserved counterparts in *P. falciparum* (E53 and R154) indeed results in the loss of *Pf*Pdx2 activity, suggesting a potential steric interference within the ammonia tunnel, thereby blocking the transmission of ammonia towards *Pf*Pdx1. Interestingly, despite the proximity of the gate to *Pf*Pdx1, the mutation of these two amino acid residues does not influence the binding capability of *Pf*Pdx2 to *Pf*Pdx1.

In conclusion, the results presented here suggest a possible path through which the plasmodial PLP synthase forms a hierarchical complex using a defined assembly sequence. Confirmation of the PLP synthase assembly path will require additional experiments to probe the dynamic behaviour of assembly. This sequence of events provides a further opportunity to interfere with the assembly of the complex and can be exploited for the development of novel chemotherapeutics to combat malaria.

## Materials and Methods

### Materials

Restriction enzymes and ligase were purchased from New England Biolabs, USA. Oligonucleotides were from Operon, Germany. The cloning vector pASK-IBA3, Strep-Tactin-Sepharose, anhydrotetracycline and desthiobiotin were from IBA (Institut für Bioanalytik, Germany). All other used chemicals were from Sigma, Germany.

### Expression and purification of the PfPdx1 and PfPdx2

*E. coli* BLR (DE3) (Stratagene, Germany) was transformed with *P. falciparum* Pdx1 and Pdx2 previously cloned into the expression vector pASK-IBA3 [6]. Single colonies were picked and grown overnight in Luria-Bertani medium containing 50 µg mL^−1^ ampicillin. The bacterial culture was diluted 1∶50 and grown at 37°C until the A_600_ reached 0.5. The expression was initiated with 200 ng mL^−1^ of anhydrotetracycline and the cells were grown for 4 h at 37°C before being harvested. The cell pellet was resuspended in 100 mM Tris-HCl, pH 8.0, 150 mM NaCl containing 0.1 mM phenylmethylsulfonyl fluoride, sonicated, and centrifuged at 50,000× g for 1 hour at 4°C. The recombinant Strep-Tag fusion protein was purified according to the manufacturer's recommendation (IBA). The eluate of the affinity chromatography was analysed by SDS-PAGE [26]. The concentration of the purified recombinant protein was determined according to Bradford [27].

### Oligonucleotides and site-directed mutagenesis of PfPdx1 and PfPdx2

Oligonucleotides were designed to replace amino acid residues in the proposed active site as well as in the interface of Pdx1:Pdx1 (Table 2). The putative active site residues of the Pdx1 domain (D26, K83 and K151) were mutated to alanine and the putative amino acid residues involved in ammonia channelling of Pdx2 were substituted by tyrosine and tryptophan (Table 2). 35 ng of the double-stranded supercoiled expression plasmid *Pf*Pdx1-IBA3 or *Pf*Pdx2-IBA3 [6] and 100 ng of mutagenic sense and antisense primers were used in a 50 µL PCR containing deoxyribonucleotides, reaction buffer, and *Pfu* DNA polymerase as described previously [28]. The cycling parameters were 95°C for 50 s, 55°C for 60 s, and 68°C for 9 min for 17 cycles. The linear amplification product was treated with endonuclease *Dpn*I (New England Biolabs) for 1 h to eliminate the parental template. A 10 µL aliquot from each PCR was used for the transformation of competent *E. coli* XL10GOLD cells (Stratagene). All mutations were verified by automatic sequencing (AGOWA, Germany). Finally, one clone of each construct was transformed for the expression in competent *E. coli* BLR (DE3) cells. The expressed proteins were purified as described above.

**Table 2:** Oligonucleotides which are used for cloning or site directed mutagenesis of *Pf*Pdx1 and *Pf*Pdx2. Mutation sites are underlined and in bold.

Name:	Oligonucleotide Sequence (5′ → 3′)
PfPdx1-D26A-S	GCTTAAAGGAGGAGTTATTATG**GCT**GTAAAAAGTGTAGAACAAGC
PfPdx1-D26A-AS	GCTTGTTCTACACTTTTTAC**AGC**CATAATAACTCCTCCTTTAAGC
PfPdx1-K83A2-S	GTTTCTATTAATGTTCTTGCT**GCT**GTTCGTATTGGTCATTTTG
PfPdx1-K83A2-AS	CAAAATGACCAATACGAAC**AGC**AGCAAGAACATTAATAGAAAC
PfPdx1-K151A-S	GAGCTTCTATGATAAGAACT**GCC**GGCGAAGCTGGTACAGGTAATATTATAG
PfPdx1-K151A-AS	CTATAATATTACCTGTACCAGCTTCGCC**GGC**AGTTCTTATCATAGAAGCTCC
PfPdx1-R85A-S	GTTCTTGCTAAAGTT**GCA**ATTGGTCATTTTGTTGAAG
PfPdx1-R85A-AS	CTTCAACAAAATGACCAAT**TGC**AACTTTAGCAAGAAC
PfPdx1-H88A-S	CTTGCTAAAGTTCGTATTGGC**GCC**TTTGTTGAAGCACAAATTTTAG
PfPdx1-H88A-AS	CTAAAATTTGTGCTTCAACAAA**GGC**GCCAATACGAACTTTAGCAAG
PfPdx1-E91A-S	GTATTGGTCATTTTGTT**GCA**GCACAAATTTTAGAAGAGC
PfPdx1-E91A-AS	GCTCTTCTAAAATTTGTGC**TGC**AACAAAATGACCAATAC
PfPdx1-RHE-AAA-S	CGTTTCTATTAATGTTCTTGCTAAAGTT**GCT**ATTGGC**GCC**TTTGTT**GCA**GCACAAATTTTAGAAGAGCTTAAAATTG
PfPdx1-RHE-AAA-AS	CAATTTTAAGCTCTTCTAAAATTTG**TGC**TGCAACAAA**GGC**GCCAAT**AGC**AACTTTAGCAAGAACATTAATAGAAACG
PfPdx1-ERR-AAA-S	GTATGTGGGTGTACAAATTTA**GGC**GCCGCTCTA**GCAGCA**ATATCTGAAGGAGCTTCTATG
PfPdx1-ERR-AAA-AS	CATAGAAGCTCCTTCAGATAT**TGCTGC**TAGAGC**GGC**GCCTAAATTTGTACACCCACATAC
PfPdx1-E107A-S	GCTTAAAATTGATATGATAGATGAAAGC**GCT**GTATTAACAATTG
PfPdx1-E107A-AS	CAATTGTTAATAC**AGC**GCTTTCATCTATCATATCAATTTTAAGC
PfPdx2-IBA3HIS-AS	GCGCGCGGTCTCAGCGCTTTAATGATGATGATGATGATGACCTGAATATTTGTAATTTTTAACC
PfPdx2-E53Y-S	GGGCTTGTAATTCCAGGTGGA**TAT**TCCACAACTGTACGTCG
PfPdx2-E53Y-AS	CGACGTACAGTTGTGGA**ATA**TCCACCTGGAATTACAAGCCC
PfPdx2-R154W-S	CTTAACAGCGGCCTGCATA**TGG**GCACCTTATATAAGAGAA
PfPdx2-R154W-AS	TTCTCTTATATAAGGTGC**CCA**TATGCAGGCCGCTGTTAAG

### Enzyme assays

The glutaminase activity of *Pf*Pdx2 was assayed in two steps, according to [5], [6], by measuring the formation of glutamate, which is subsequently converted to 2-oxoglutarate by glutamate dehydrogenase with acetylpyridine adenine dinucleotide (APAD) as co-substrate. For activity the enzyme complex consisting of Pdx1 and Pdx2 is required [6]; therefore, both enzymes were mixed in an equimolar ratio (total amount 30 µg). The assay was performed in 50 mM Tris-HCl, pH 8.0 in the presence of 10 mM glutamine in a total volume of 300 µL at 37°C for 20 min. The enzymatic reaction was stopped by boiling for 1 min. A 50 mM Tris-HCl, pH 8.0 buffer containing 1 mM EDTA, 0.5 mM APAD and 7 units of glutamate dehydrogenase was added to a final volume of 1 ml and incubated for up to 90 min at 37°C. Finally, the samples were centrifuged for 1 min at 14,000× *g* and the absorbance of the supernatant was determined at a wavelength of 363 nm. The specific activity was calculated with the molar extinction coefficient of APADH (reduced form of APAD) of 8900 M^−1^ cm^−1^.

For the synthesis of PLP, the enzymes Pdx1 and Pdx2 are required. The reaction was performed in the presence of the substrates 0.5 mM ribose 5-phosphate, 1 mM glyceraldehyde 3-phosphate and 10 mM glutamine in a buffer containing 100 mM Tris-HCl, pH 8.0, and 150 mM NaCl [24]. PLP biosynthesis by Pdx1 alone was carried out under equivalent conditions; however glutamine was replaced by 10 mM ammonium salt. The total volume of 1 mL per reaction was incubated for 30 min at 37°C. Subsequently, the supernatant was analysed at 414 nm for the formation of a Schiff base between PLP and the Tris base using a spectrophotometer [7], [8].

### Co-purification experiments of PfPdx1 and PfPdx2

The plasmodial proteins were recombinantly expressed as described above. After sonication and centrifugation, the supernatants of the respective Pdx1 wild-type or mutant proteins were combined with the supernatants of the *Pf*Pdx2 expression. For co-purification the Strep-tag encoded by the pASK-IBA3 expression vector was substituted by a 6× His-tag using a PCR reaction containing the oligonucleotides *Pf*Pdx2-IBA3-S and *Pf*Pdx2HIS-IBA3-AS according to [6]. Subsequently, the mixture of *Pf*Pdx1 and *Pf*Pdx2HIS was purified via Strep-Tactin affinity chromatography as described above. For visualizing the co-purification, Western blot analyses were performed employing a monoclonal Strep-tag II antibody (IBA) at a dilution of 1∶20000 and a secondary anti-mouse horseradish peroxidase labelled goat antibody (BioRad, Germany) at a dilution of 1∶20000. The *Pf*Pdx2HIS protein was detected by the HIS-Probe-HRP reagent (Pierce, USA) at a dilution of 1∶5000. The hybridization signals were visualised on X-ray films (Retina, Germany) using the ECL plus detection system, according to the manufacturer's instructions (GE Healthcare).

### Determination of the oligomeric state of PfPdx1

In order to investigate the complex formation of *Pf*Pdx1, the Strep-tagged protein was purified as described above. Subsequently 100 µg of the protein were separated by gel filtration on a Superdex 200 10/30 column (GE Healthcare). The elution buffer contained 100 mM Tris-HCl, pH 8.0, 150 mM NaCl with and without 10 mM glutamine, glutamic acid, asparagine or alanine, respectively. A miniDAWN Tristar (Wyatt Technologies, USA) was connected immediately downstream of the separation media and used to collect static light scattering (SLS) data [29]. The SLS data were analyzed using the package ASTRA, based on the absorption coefficient for *Pf*Pdx1 of 19855 M^−1^ cm^−1^ and the molecular mass of 34.2 kDa.
